# The return home model: design and implementation of a geriatric home-care model for long-term care eligible older adults

**DOI:** 10.1186/s13584-025-00719-y

**Published:** 2025-09-18

**Authors:** Inbal Mayan, Kristine Yaffe, Adam J. Rose, Isabel E. Allen, Gila Yakov, Glynis Katz, Irit Fischer-Reif, Ron Sabar

**Affiliations:** 1 Sabar Health, HaMahshev St 3, Netanya, Israel; 2 Global Brain Health Institute (GBHI), University of California San Francisco, San Francisco, CA USA; 3 Departments of Psychiatry, Epidemiology & Biostatistics, and Neurology, University of California, San Francisco, CA USA; 4 Faculty of Medicine, School of Public Health, Hebrew University of Jerusalem, Jerusalem, Israel; 5 Department of Epidemiology & Biostatistics, School of Medicine, University of California San Francisco, San Francisco, CA USA; 6 Department of Health Systems Management, The Max Stern Yezreel Valley College, Yezreel Valley, Israel; 7 JDC-Eshel, Jerusalem, Israel

**Keywords:** Home-care geriatric model, Aging in place, Dependent older adults, Long term care placement

## Abstract

**Background:**

Most older adults prefer to “age in place” within their communities. This preference cannot always be honored and dependent older adults may transfer to a long-term care facility. The Return Home is an Israel Ministry of Health initiated care model designed to prevent or delay a transfer of the dependent older adult to a long-term facility. The intervention team included a physician, nurse, social worker, occupational therapist, physical therapist, and a dietician, all participating in in-home visits. This study’s aim was to examine the Return Home model’s feasibility to prevent long-term care placement in a complex, dependent geriatric population.

**Methods:**

We analyzed data from the electronic medical record (EMR) of the provider. Participants were recruited by the Israeli Ministry of Health from July 2021 to November 2022 at the time of hospital discharge. Caregiver input was obtained from interviews at the beginning and end of the one-year intervention.

**Results:**

138 patients were enrolled in the intervention. 86 (62%) completed the intervention in their homes, 39 (28%) died during the intervention, 5 (4%) were transferred to a long-term facility, 8 (6%) were dis-enrolled. Prescription medication usage declined by 0.79 medications per person on average. Forty patients had pressure ulcers at the time of admission; all of these ulcers healed during the program, after an average time of 1.5 months. Caregiver burden measured by the Zarit score, declined from 20.9 to 9.7, t (156) = 11.88, *p* < 0.001.

**Conclusions:**

The Return Home intervention demonstrated the feasibility of preventing or delaying long-term care placement for a complex, dependent geriatric population. Further evaluation is needed to determine effectiveness and inform broader implementation.

## Background

In Israel, approximately 2% of older adults (age 65+), or over 50,000 people, reside in long-term care (LTC) facilities [1]. While Israel has a large young population, due mainly to high fertility rates, the number of older adults who are functionally dependent is growing rapidly and their medical needs are imposing a growing burden on the health system [2]. Those who are dependent for activities of daily living (ADL), as well as individuals diagnosed with Alzheimer’s disease (AD) and AD related dementias (ADRD), are often placed in facility-based long-term care (LTC) due to significant clinical and functional limitations [3]. The decision to transition an older adult into a LTC facility is multifaceted and complex, influenced by various factors including the overall capability of caregivers, cultural beliefs, the patient’s health status, the capabilities of the healthcare system, and the suitability of the elder’s home environment [4]. This decision is typically made as chronic comorbidities become more severe, functional impairments worsen, neuropsychiatric symptoms increase, and the home environment becomes less conducive to managing the deteriorating medical situation [5–7]. 

Most older adults in Israel, and elsewhere, express a preference to “age in place” [8, 9]Aging in place is defined as the ability to live safely, independently, and comfortably in one’s own home and community, regardless of age, income, or ability level [10]. The benefits of aging within the community, both for the older adults and the community itself, are well-documented and include a strengthened sense of community and belonging, a deeper connection to the surrounding environment, and positive impacts on the elder’s quality of life [11]. Given these advantages, it is important that health policymakers and clinicians advocate for and support this preference. Furthermore, the high costs associated with LTC facilities, which are borne by the families of dependent individuals and by the state, underscore the need to find ways to support such a choice as often as possible [2]. In Israel, the average cost of LTC facility care is approximately 115 USD per day (equivalent to over 3,400 USD per month), placing a substantial financial burden on both families and the health system [12]. 

There have been previous initiatives to try to improve the quality of home care for vulnerable older adults. These initiatives, which mostly occurred in developed countries, focused primarily on symptom management or chronic disease management, rather than on delaying long-term care placement [13–15]. Other models have been developed to change the culture of nursing homes and LTC facilities in order to make them more community-like, by allowing plants and animals in the Eden Alternative Model [16] or giving the residents more choice in their day-to-day activities in the Green House Model [17]. While functional status is a central eligibility criterion for home health care globally, including in Medicare eligibility in the United States [18], across OECD countries [19], and in international LTC frameworks [20], we have not found published interventions that aim to fully replace LTC facility placement by providing facility-level interdisciplinary care within patients’ homes.

Based on the need to address these pressing issues, the Israeli Ministry of Health developed the “Return Home Model”, a home-based care model designed for dependent older adults. This model aims to serve as an alternative to LTC placement, while maintaining quality of care and supporting caregivers. It also seeks to align the incentives of patients, families, healthcare providers, the health maintenance organizations, and the government of Israel, which is the final common payer in the system. This program was subjected to a nationwide one-year pilot study.

Here, we present a descriptive analysis of enrollees in the “Return Home Program” that were treated by Sabar Health, a hospital at home (HaH) provider, and their outcomes after one year in the program, at which time the pilot program ended. The primary aim of this study is to establish the feasibility of this new care model as a program aiming to provide LTC in the home and not in a healthcare facility. Secondarily, this study examines the impact of the program on clinical outcomes, caregiver burden, and caregiver depression.

## Methods

All patient-level data was extracted from the institutional electronic medical record (EMR). Consent was provided by the patients where applicable, and by a legally authorized representative when patients could not give consent. Caregivers were surveyed over the phone at the beginning and end of the intervention. The protocol and consent materials were approved by the Institutional Review Board of Sheba Medical Center.

**Fig. 1:**
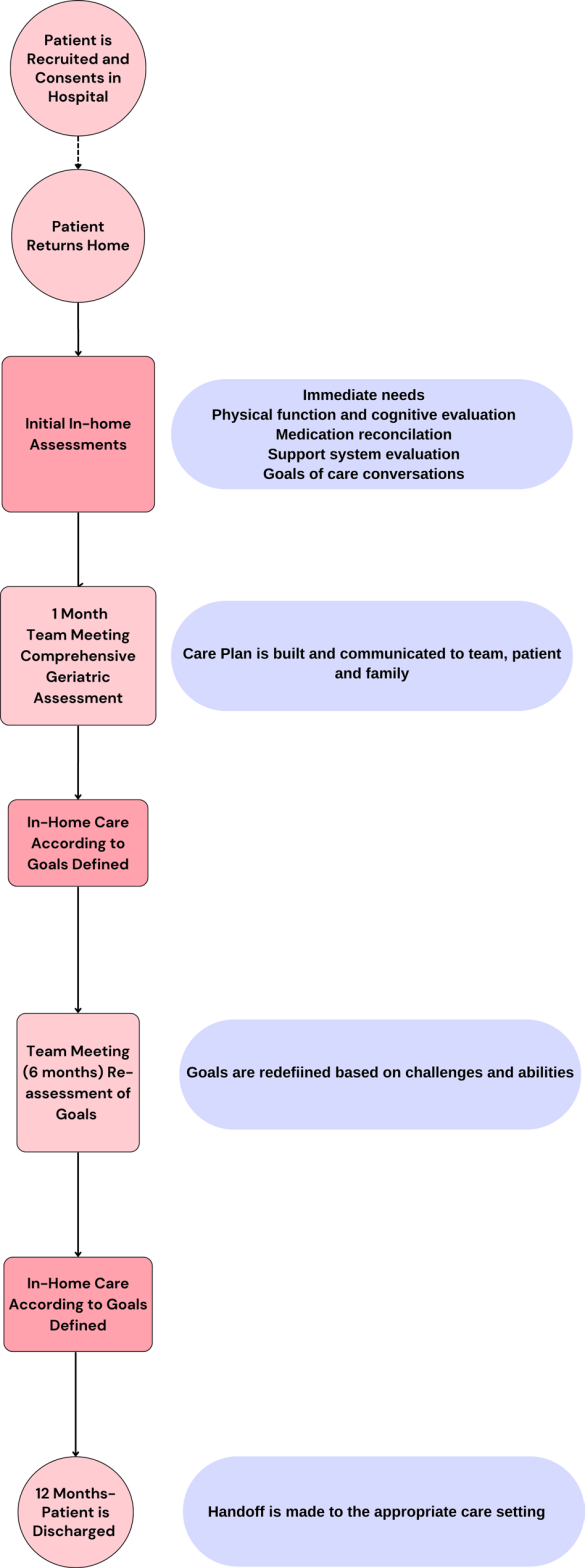
Overview of “The Return Home Model” flow. The program lasted for a one year period that started as the patient returned home from an acute hospitalization. The patient was discharged back to usual care at the end of the year. Main points in the course of the program shown here

Overview of the model (Fig. 1).

The Return Home is an Israeli Ministry of Health initiative, which enrolled patients currently admitted to acute care wards in general hospitals, who are about to transition to a long-term facility. Patients were offered the chance to instead receive medical, nursing, and social care in their homes. Exclusion criteria were minimal and are described in detail below. Sabar Health, a leading HaH provider in Israel, was chosen to lead and implement this effort.

The program was administered in patient’s homes in the geographical center and north of Israel. The intervention consisted of home visits by medical professionals at predetermined intervals. All team members underwent an initial orientation to the Return Home model, including interdisciplinary care planning, home safety assessments, and caregiver engagement strategies, and received ongoing training under geriatrician supervision throughout the program. Visits were performed by a physician, a nurse, a social worker, a physical therapist, an occupational therapist, a dietitian, and if deemed necessary by the care team, a speech pathologist. On average, patients received frequent visits early in the program, including approximately 12 nurse visits and 8 physician visits in the first month. Visit frequency tapered over time based on patient needs, with monthly nurse visits and occasional physician follow-ups by the final months. Physical and occupational therapy, social work, and dietitian visits were also front-loaded in the first months to establish care plans and then provided as needed thereafter. A care navigator, a nurse by training, oversaw communication among the care team, family and patients. Each full-time care navigator took on a 50-patient case load. The care navigator visited the home of the patient at admission to the program and then continued communication via phone as needed. A geriatric physician led the care teams and was consulted on medical matters by the physicians and nurses.

Each patient’s care began with the team performing a comprehensive geriatric assessment and building a care protocol. Thereafter, changes in the protocol were made throughout the year by the geriatric physician together with the care navigator, according to patients’ and caregiver needs and the changing situation.

Care teams had two scheduled meetings during the program, one after a month where the care plan was finalized, a second after six months where it was reevaluated. Other meetings could be called according to need. When the medical condition worsened, an internal escalation protocol was initiated and included consulting the geriatric physician lead, a pharmacist when necessary, and the Ministry of Health officials when relevant.

Patients were discharged from the program after a year had passed from enrollment, as a part of the program protocol. At that time, patients reestablished care with their primary care physician or were referred to home hospice or other care based on their needs and preferences.

Patient baseline health complexity and caregiver involvement varied across participants and may have influenced individual outcomes.

Patient Identification and Enrollment (Inclusion and Exclusion Criteria).

138 patients were enrolled in the intervention from July 2021 to November 2022. Participants and families were approached by social workers in acute wards in 12 hospitals around the country and asked if they wanted to join the program. Social workers approached participants and families only if they met criteria for long term care placement and if that process had already been initiated by the families. Eligibility criteria included age 65 and older, willingness to participate, dependent ADL status, a family caregiver willing to communicate with the care team, and a patient who was already in the process of being referred to long-term care. All participants could either be described as having advanced dementia, severe frailty, or both. There were no other exclusion criteria. All eligible patients were enrolled.

### Measures

The primary outcome of this study was preventing transition to a LTC facility within the year of the intervention (i.e., for one year after the date of enrollment into the program). We used electronic medical records (EMR) to track time in the intervention and outcomes of transitions in care, including disenrollment due to unforeseen circumstances such as hospitalization or death.

Secondary outcomes included changes in the number of medications, prevalence of pressure ulcers and time to healing, delirium prevalence and time to delirium subsiding, caregiver burden, and caregiver depression scores.

#### Patient outcomes

Number of medications included all medications taken regularly, such as daily or weekly, but not skin creams or medicines taken as needed. The number of medications was assessed at admission and at discharge. All patients were assessed on admission for the presence of pressure ulcers. Patients were visually examined at least monthly, while those with known ulcers were assessed by a nurse at least weekly. An incident ulcer was defined as the appearance of stage II, III, or IV ulcer that was not previously noted. Delirium was assessed on admission by a physician using a clinical assessment and the short CAM score [21]. In patients where delirium was present, assessments continued daily by phone and in person at least weekly until delirium subsided.

*Caregiver Outcomes*: Caregiver burden was assessed using the short Hebrew version of Zarit Burden Interview (H-ZBI) [22]. The short version of the ZBI comprises 12 questions: each question is scored on a five-point Likert scale ranging from 0 (never) to 4 (almost always) with cumulative score ranging from 0 to 48. A higher score indicates a greater sense of burden with 0–9 indicating no to mild burden, 10–20 moderate burden, and above 20 severe burden [23]. 

Caregiver depression was assessed at the beginning of the intervention and its end by phone interviews using the Hebrew validated version of the Patient Health Questionnaire (PHQ-9) [24]. This scale is based on the nine criteria for diagnosing depression in the Diagnostic and Statistical Manual of Mental Disorders IV (DSM-IV). Each item is scored on a scale of 0–3 (0 = completely absent, 1 = some days, 2 = more than seven days in the past month, 3 = almost every day), with a total score of 0–27. Higher scores indicate more severe depression [25]. 

#### Calculation of costs

Costs of the program were calculated based on the contract between the Ministry of Health and Sabar Health. Costs were compared with the known costs of long-term care in a facility, as published in the Ministry of Health price list [12]. 

#### Data collection

Data on patient outcomes was collected retrospectively from electronic medical records in April 2024, after the end of the pilot program. A survey of caregivers was conducted by phone at the beginning and end of the intervention, and at the one-year mark when the patient did not remain in the program for a full year. Phone surveys included a PHQ9 and Zarit score. Multiple phone call attempts were tried at different times of day to maximize participation.

#### Statistical analysis

Data analysis was performed in May 2024 using Stata 18.1 (StataCorp, College Station, TX). Initially, we summarized descriptive statistics to describe our sample’s demographic characteristics (means and standard deviations for continuous variables and frequencies and percentages for categorical variables). For the primary outcome (remaining in the program until the end of the program) we performed a Kaplan-Meier 12-month survival analysis. All patients in the study were enrolled in the program, and therefore, the main analysis compared the primary outcome between patients with dementia and those who are frail. For Secondary outcomes we used student’s independent groups t-test to compare between different groups.

## Results

138 patients were enrolled in the intervention (56.0% female, average age 86.7). 92 (67.0%) of patients enrolled had an advanced dementia diagnosis and 46 (33.0%) were highly frail with no dementia diagnosis. Complete follow-up data was available for all patients regarding their final disposition and patient-relevant outcomes. However, only 79 caregivers (57.0%) completed both beginning and end of intervention interviews, limiting our ability to compare caregiver burden at program enrollment vs. at program discharge. Of those caregivers who did respond, (73.0% were female, with an average age of 60. 65 (82.3%) of caregivers were the children of the patients. Baseline characteristics for participants and caregivers are shown in Tables 1 and 2.

**Table 1:** Patient baseline characteristics (*n* = 138)

Demographic Characteristics	Proportion	Frequency
*Sex*		
Female	56%	78
Male	44%	60
*Age*		
65–74	9%	12
75–84	36%	49
+ 85	56%	77
*Enrolling condition*		
Dementia	67%	92
Frailty	33%	46

**Table 2:** Caregiver baseline characteristics (*n* = 79)

Demographic Characteristics	Proportion	Frequency
*Sex*		
Female	73%	58
Male	27%	21
*Age*		
< 40	3%	2
40–50	36%	9
50–60	38%	30
60–70	29%	23
70–80	13%	10
> 80	6%	5
*Relationship with patient*		
Daughter	57%	45
Son	25%	20
Spouse	17%	13
Granddaughter	1%	1
*Education level*		
8 Years or less	2%	2
Highschool	27%	21
Professional college (2 years)	8%	6
College	63%	50

### Primary outcome

**Fig. 2:**
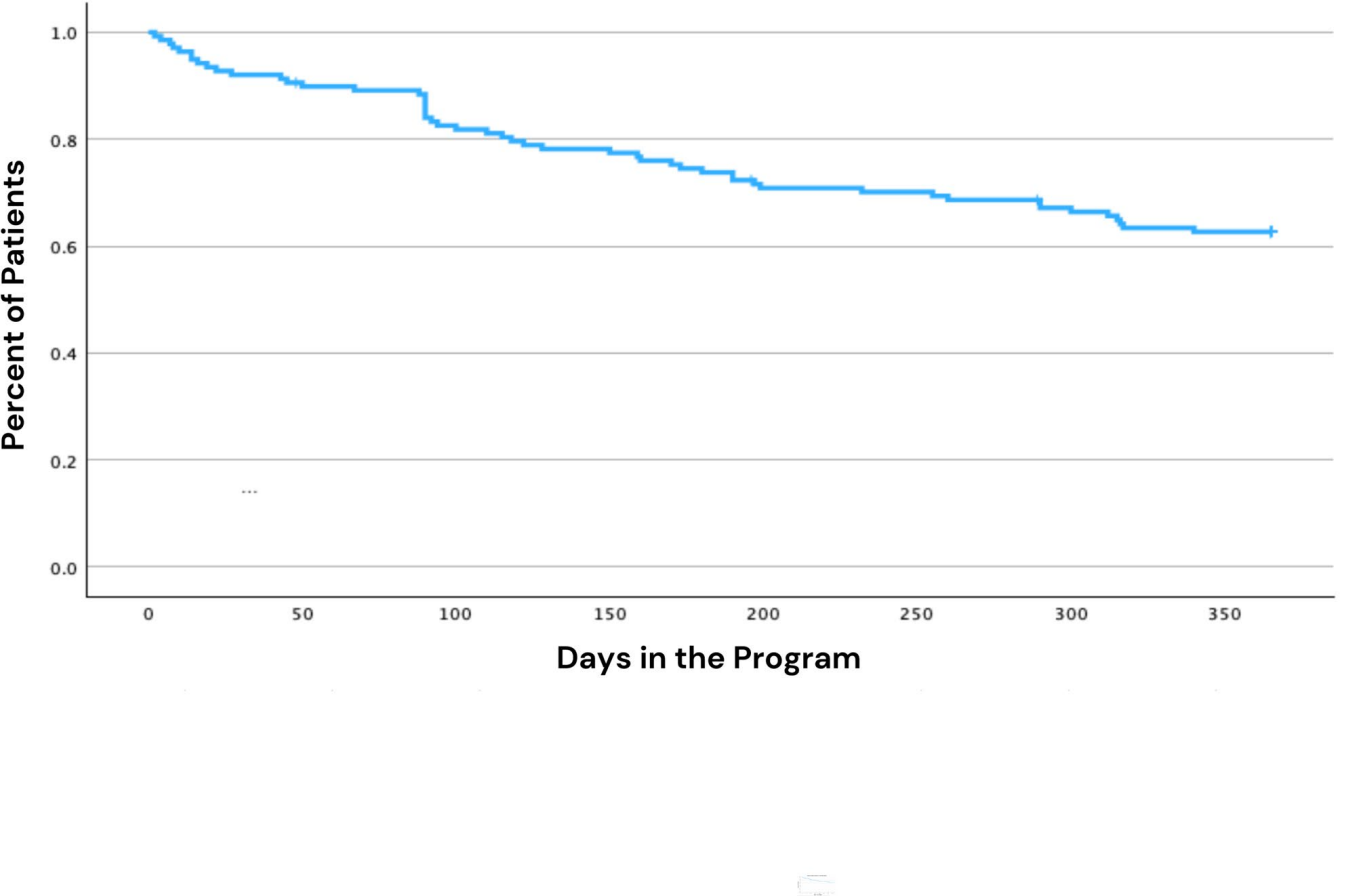
Kaplan Meier plot, number or days in intervention.A Kaplan- Meier plot showing the number of days patients remained within “The Return Home Model” program

86 (62.0%) of patients completed the full one-year intervention in their homes, 39 (28.0%) died during the intervention, 5 (4.0%) were transferred to a long-term facility, and 8 (6.0%) were disenrolled for other reasons, such as being sent to the hospital. The average number of days in the intervention was 279 (SD = 10.9) (Fig. 2). No statistically significant differences in total days in the program were found between the dementia and non-dementia group, by the sex of the patient, or by occurrence of delirium.

### Secondary outcomes

The number of medications per patient declined by 0.8 per patient, on average, during program participation, from an average of 7.5 medications at the beginning to 6.7 at the end (*p* < 0.001). In stratified analyses by sex, presence of delirium, or the diagnosis of dementia, no statistically significant between-group differences were seen regarding this decrease in the number of medications.

40 patients had pressure ulcers on admission. All 40 pressure ulcers healed during the patient’s time in the program, after an average of 1.5 months (range: 0.5 to 2.5 months). No new pressure ulcers were noted during program participation. 27 patients were admitted to the program with delirium. Delirium resolved for all these patients, after an average of two weeks (range: 1 to 10 weeks). Of these 27 patients with delirium on admission, only 3 (11%) received psychoactive medications. No new patients developed delirium while enrolled in the program.

Caregiver burden, as measured by the Zarit score, declined from 20.9 (high burden) to 9.7 (low burden) from admission to the end of the program, t (156) = 11.88, *p* < 0.001 for difference. The analysis of the Zarit score was repeated after stratifying based on the presence or absence of a dementia diagnosis, with no statistically significant difference between groups. The PHQ-9 score of caregivers did not change significantly over the course of the program.

Costs of the program were 35 USD per day which is lower than long term care which costs approximately 115 USD per day (70% cost reduction).

## Discussion

The Return Home program was pilot tested by Israel’s Ministry of Health as an alternative to transferring older adults from acute care hospitals to long-term care facilities. In this feasibility study, 138 patients needing assistance with basic ADLs were enrolled for up to one year. The intervention successfully prevented LTC placement for 133 participants and cost 70% less than traditional facility-based care. We also observed promising results in patient outcomes (medication reduction, pressure ulcer healing, delirium resolution) and caregiver burden reduction. These findings suggest the intervention is a feasible alternative to facility-based LTC and may support patients’ preference to remain at home. However, further evaluation is needed to confirm effectiveness and generalizability.

Programs like PACE in the United States [26], which integrates medical and social care to help older adults remain in the community, and Buurtzorg in the Netherlands [27], which uses self-managed nursing teams for home-based care delivery, have shown success in supporting aging in place. However, the Return Home program differs by specifically targeting individuals already approved for LTC placement, providing comprehensive interdisciplinary care at home as a full replacement for institutionalization.

This study has several notable strengths. This is a novel study, in that we showed that a holistic approach to providing longitudinal in-home care that is not based on any particular underlying diagnosis is feasible. We are not aware of any previous published studies of such a program. We also demonstrated the feasibility of an ethical model of care that aligns with older adults’ wishes for medical care and aging within their communities. Our approach was based on providing care in the community with a holistic care approach. This approach differs from the steps taken recently by the the Centers for Medicare & Medicaid Services in the United States, that promote interventions that support patients within their homes based on their diagnosis and not on their functional status. Dementia focused home based support is the goal of Guiding an Improved Dementia Experience Model that began implementation in July 2024 for patients with Dementia and their caregivers [27]. Other interventions that have been studied thus far were also diagnosis-specific for either cancer patients [28, 29]or end stage organ failure patients.[13, 14, 30, 31] Our model was able to accommodate patients with those diagnoses but was not specific to any of them.

Several aspects of the intervention design support potential implementation elsewhere. The model’s flexibility allowed care to be tailored to each patient’s changing needs, facilitated by care navigators and interdisciplinary team oversight. While the pilot was limited to one year due to funding design, a considerable proportion of patients (86, or 62.0%) were doing well in the program at one year after enrollment, and there was no medical reason why they could not have continued for a period exceeding one year.

It is important to acknowledge key limitations of our study. First, it lacked a control group, limiting our ability to infer causality. Second, participants were identified by hospital social workers once LTC placement was already underway, potentially introducing selection bias if families were particularly motivated to keep their loved one at home. Third, only 57% of caregivers completed the exit interviews, limiting generalizability of caregiver outcomes. Additionally, as a single-provider observational feasibility study, results may not fully translate to other settings or populations without adaptation.

These findings have important implications for future policy and research. Looking ahead, national adoption of the Return Home model would require sustained funding streams, sufficient trained interdisciplinary workforce capacity, and robust coordination between hospital discharge planners and home care providers. Ensuring quality and safety at scale may necessitate standardized protocols, data systems for monitoring outcomes, and integration with primary care services. Further research is needed to assess long-term patient and caregiver outcomes, cost-effectiveness, and optimal implementation strategies in diverse settings.

## Conclusions

Caring for dependent older adults imposes a significant and growing burden on health systems, families, and patients themselves. Facility-based long-term care is an expensive and complex solution, particularly as populations age rapidly. Therefore, alternative care models are urgently needed. This feasibility study demonstrated that a geriatric, ‘diagnosis-agnostic’ home care model may be a viable alternative to facility-based long-term care for dependent older adults in Israel. However, further evaluation is required to confirm effectiveness, assess cost-effectiveness at scale, and determine optimal implementation strategies before broader adoption. Such models have the potential to support aging in place and reduce reliance on institutional care for those who prefer to age in place and remain at home.

## Data Availability

The datasets analysed during the current study are not publicly available as they are a part of a medical record, but the anonymized data is available from the corresponding author on reasonable request.

## Abbreviations

EMR: Electronic medical record
ADL: Activities of daily living
LTC: Long term care
AD: Alzheimer’s dementia
ADRD: Alzheimer’s dementia related dementias
HaH: Hospital at home
ZBI: Zarit Burden Interview
PHQ-9: Patient Health Questionnaire
